# Effects of financial incentives for treatment supporters on tuberculosis treatment outcomes in Swaziland: a pragmatic interventional study

**DOI:** 10.1186/s40249-015-0059-8

**Published:** 2015-06-05

**Authors:** Merav Kliner, Mamvura Canaan, Sifiso Zwide Ndwandwe, Fred Busulwa, William Welfare, Marty Richardson, John Walley, John Wright

**Affiliations:** The Clinic Group, Matsapha Health Care, PO Box 1075, Matsapha Manzini, Swaziland; Family Life Association of Swaziland, PO Box 1051, Manzini, Swaziland; Good Shepherd Hospital, PO Box 2, Siteki, Swaziland; Manchester Academic Health Science Centre, University of Manchester, Oxford Rd, Manchester, M13 9PL UK; Cochrane Infectious Diseases Group, Liverpool School of Tropical Medicine, Pembroke Place, Liverpool, Merseyside L3 5QA UK; Nuffield Centre for International Health and Development, University of Leeds, 101 Clarendon Road, Leeds, LS2 9LJ UK; Bradford Institute for Health Research, Bradford Royal Infirmary, Duckworth Lane, Bradford, BD9 6RJ UK; Mersey Deanery, Summers Road, Liverpool, Merseyside L3 4BL UK

**Keywords:** Tuberculosis, Treatment success, Swaziland, Africa

## Abstract

**Background:**

Swaziland has the highest national incidence of tuberculosis (TB) in the world, with treatment success rates well below the 85 % international target. Treatment support as part of comprehensive TB services is a core component of the Stop TB Strategy. This study investigated the effects of financial incentives for treatment supporters on TB treatment outcomes in Swaziland.

**Methods:**

This was a controlled study that compared treatment outcomes for patients with a treatment supporter who received or did not receive a financial incentive.

**Results:**

The intervention group had a higher chance of treatment success as compared with the control group: 73 % (95 % confidence intervals [CIs] 66–80 %) versus 60 % (95 % CIs 57–64 %), respectively, *p* = 0.003. This improvement remained significant when treatment success rates were adjusted for differences in baseline characteristics, with the effect of incentivised treatment supporters on treatment outcomes having an odds ratio (OR) of 1.8. There was also a significant improvement in the death rate in the intervention group, as compared with the control group (10.6 versus 23.5 %, *p* = <0.001).

**Conclusion:**

Incentives provided to TB treatment supporters appear to significantly improve TB treatment outcomes. Incentivising treatment support may be appropriate as an effective addition to support and supervision measures (199 words).

**Electronic supplementary material:**

The online version of this article (doi:10.1186/s40249-015-0059-8) contains supplementary material, which is available to authorized users.

## Multilingual abstracts

Please see Additional file 1 for translations of the abstract into the six official working languages of the United Nations.

## Background

Adherence to drug treatment and appointments for tuberculosis (TB) is critical to prevent drug resistance. The Stop TB Strategy places a significant emphasis on patient support and supervision as part of the comprehensive TB services, including directly observed therapy (DOT) [1]. However, evidence about the effectiveness of DOT is limited and this therapy can be a considerable burden for patients and healthcare services, as it requires staff to visit patients and observe them taking medication each day [2]. The World Health Organization (WHO) recommends that patients get themselves a treatment supporter, who may either be a health worker or a trained and supervised member of the community or family, on diagnosis [3]. The treatment supporter should share responsibility for completion of treatment and should provide treatment supervision, which may include DOT as well as social and psychological support.

Incentives for provision of treatment support, such as food and travel costs for appointments, may be appropriate in order to support a patient with their treatment. A 2011 Cochrane Review of randomised controlled trials (RCTs) investigating the impact of material incentives in the investigation or treatment of TB, identified 11 studies [4], with the majority of these conducted in the USA among vulnerable groups such as drug users, homeless people and prisoners. Only one study of 265 patients in Timor-Leste considered the effects of financial incentives on treatment outcomes; the study didn’t find any effect on the outcomes [5]. Another study, not included in the Cochrane Review, about monetary incentives for TB treatment in Ecuador, found that the national programme of financial incentives reduced default rates from treatment from 26 to 9 % [6], and alleviated the financial burden of treatment for patients. A pragmatic cluster RCT in South Africa, which compared treatment outcomes among patients who received a voucher and those who didn’t, found no difference in treatment outcomes between the two groups [7]. A controlled before-and-after study of rural-to-urban migrants in China found a significant increase in treatment completion in those receiving financial incentives [8]. No studies were identified that focused on incentives been given to treatment supporters rather than directly to the patients.

Swaziland is a landlocked country situated in Southern Africa, bordering Mozambique and South Africa. The country’s key indicators are outlined in Table 1. Swaziland has the highest national incidence of TB in the world at 1382 per 100,000, with rates increasing 5-fold over the past 10 years [9]. The TB incidence is fuelled by poverty, poor housing, overcrowding, malnutrition and a poor healthcare infrastructure, but is mainly related to the country’s HIV burden, which, with a prevalence of 17 %, is also one of the highest in the world [10]. In 2013, 74 % of new TB cases were co-infected with HIV [10]. The international target, for TB treatment success was originally set by the World Health Assembly in 1991 and was incorporated into the Millennium Development Goals (MDGs). TB treatment success in Swaziland is 72 %, considerably lower than the international target of 85 % [10,11]. Multidrug resistant (MDR) TB rates are high at 8 and 34 % of new and re-treated patients, respectively [12].

**Table 1:** Key indicators for Swaziland [22]

Total population (2013)	1,250,000
Gross national income per capita ($, 2013)	6,220
Literacy rates in over 15s (%)	87.8
Life expectancy at birth m/f (years, 2012)	52/55
Prevalence of HIV among adults aged 15–49 (%)	27.4 (26.6–28.1)
Deaths due to HIV/AIDS per 100,000 population	443
Incidence of TB (per 100,000 population per year)	1382 (1220–1541)
Case detection rate (all forms TB) (%)	38 (34–44)

The National TB Programme and regional government offices manage the TB burden in Swaziland. There are a number of government and non-governmental hospitals and clinics that provide diagnosis and treatment for the majority of TB patients. Patients are diagnosed with TB on sputum smear and culture. After initiation on TB treatment, if patients do not improve and sputum fails to convert to smear negative, the patient is referred to the national TB hospital. This hospital diagnoses, admits and treats MDR TB patients. Community-based organisations (CBOs) provide support to the community on a wide range of social and health issues, including support for TB treatment.

To improve treatment success rates and consequently achieve the international target, a number of interventions are being tested in the country, including comprehensive provision of treatment support for TB. The Swaziland Tuberculosis Emergency Response Plan recommends DOT for all TB patients, and provides patient support and incentives for treatment support [13]. Across Swaziland, incentives were provided to 12 CBOs to support DOT through external funding from The Global Fund to Fight AIDS, Tuberculosis and Malaria (Global Fund), via the National TB Programme. The CBOs were advised to spend the funding as required in order to increase treatment completion. A multi-centre study, which includes treatment outcomes for patients supported across all 12 CBOs, including the Good Shepherd Hospital (GSH), will be published soon. The multi-centre study investigates the impact of different methods of using financial incentives, including the provision of financial incentives directly to patients.

This study presents the findings from the GSH TB Programme, the CBO supporting the largest number of patients in the multi-centre study. This CBO chose to use the funding to provide incentives to treatment supporters, as one method of providing financial incentives for the programme. This single-centre study aimed to assess the impact on treatment outcomes of providing financial incentives directly to TB treatment supporters.

## Methods

We adopted a pragmatic controlled interventional study design to investigate the effects of incentives for treatment supporters on treatment outcomes in patients diagnosed with TB at the GSH in Lubombo, a rural district in Swaziland, which has a population of over 200,000. The GSH is the regional hospital in Lubombo and is the diagnostic centre for TB.

### Study population

We included all patients diagnosed with TB between 1st January 2010 and 30th September 2011 at the GSH.

Patients were divided into the intervention and control groups, which was determined depending on where they lived. As this was a pragmatic study, the GSH already had a small number of volunteer community support workers (CSWs) who, at that time, provided informal treatment support to HIV patients. Thus, those who lived in the same community as a CSW were included in the intervention group. Those who lived in other communities were included in the control group (standard care).

### Intervention

Both the control and the intervention groups received treatment support from a trained treatment supporter. This included: DOT, appointment reminders, support getting to clinics, and social and psychological support. Following the national guidelines, all TB patients were asked to identify and bring a family or community member to the appointment at which TB treatment was initiated. The control group received treatment support from their family or community member. The intervention group received treatment support from a CSW, who was given a monthly financial incentive for each patient under his or her care while the patient remained on TB treatment. This small incentive was to cover travel for the CSW to the clinic with (or on behalf of) the patient and to provide food or other supplies to aid the patient’s recovery. In essence, this was a minimal stipend to show treatment supporters appreciation for expending resources as they carried out their work.

The CSWs were originally recruited as HIV community workers in 2005 and received initial training in HIV/AIDS and communication skills in a 2-day workshop. They then delivered weekly support groups for HIV patients within their respective communities. They continued to work on a voluntary basis with the GSH from 2005 until after the study period, with regular monthly training sessions. They were chosen to be involved in the intervention due to their close working relationship with the hospital.

The family/community member and the patient received the same education and training by the nurse in the treatment clinic. The CSWs received the same TB treatment support training as the family/community member prior to the start of the intervention. The patients themselves did not receive a financial incentive as the treatment supporter provided the intervention. The CSWs received payment equivalent to US $5.75 per month for each patient and an additional payment equivalent to US $34.40 for each patient who completed treatment or was cured after 6 months. This was determined based on the overall funds available and weighted to incentivise treatment completion.

### Outcome measures

Following the WHO definitions for TB treatment outcomes [14], the primary outcome measure was the proportion of patients with treatment success: treatment completion or cure. Secondary outcomes were the rates of other treatment outcomes (treatment completion, cure, default, death, treatment failure and transferred out) in the intervention group compared with the control group.

### Data collection and analysis

We collected treatment outcomes from the TB register at the GSH (hard copy) and identified those receiving the intervention through their unique national TB number. A data entry clerk entered the data into Microsoft Access. Baseline characteristics were limited to the data that were routinely collected by the TB register. This included: age, sex, TB category, site of disease, HIV status and whether a patient was initiated on HIV treatment. We compared the baseline characteristics of the intervention and control groups using the chi-square test.

We compared the proportion of patients who had treatment success in the intervention and control groups using a chi-squared test for Fisher’s exact test of independence, where appropriate. In order to estimate the effect of having an incentivised treatment supporter on the success of treatment, a logistic regression model was implemented, utilising forward stepwise selection to identify covariates one by one that had to be included in the model, based on the results of the likelihood ratio test. The covariates entered into the model were pre-specified, and it was hypothesised before building the model that they may have a confounding effect. The model produced estimates of the coefficients and the corresponding standard errors for each covariate included in the model, alongside a Wald chi-square value and corresponding *p*-value, which were used to test the null hypothesis that the coefficient’s true value is zero. Confidence intervals (CIs) of 95 % were used to determine whether coefficients are significantly different from 0. Odds ratios (ORs) were also generated for each covariate. Analysis was undertaken using SPSS Statistics version 19.

### Sample size calculation

A treatment success rate of 72 % was assumed, which was the national treatment success rate in 2010 when this study was undertaken [15]. We estimated that a 13 % increase in treatment success would be relevant for the public health programme, as it would increase treatment success to the Stop TB target of 85 %. Using alpha of 0.05, power of 0.9 and 900 patients in the control group, a sample size of 92 in the intervention group was required.

### Ethics

This study was approved by the Swaziland Ministry of Health Ethics Committee, reference MH-599C.

## Results

Of the 1077 patients initiated on TB treatment between 1st January 2010 and 30th September 2011 at the GSH, 916 patients received standard care (control group) and 161 patients received support from treatment supporters who were given financial incentives (intervention group). The baseline characteristics of the two groups are presented in Table 2. There were no statistical differences for age and gender between the two groups, but they did differ in HIV status, with the intervention group having a lower proportion of patients with co-infection (77.4 versus 68.9 %, *p* = 0.021).

**Table 2:** Baseline characteristics of patients

	Control (*N*, %)	Intervention (*N*, %)	*p*-value
Total	916	161	
Male sex	457 (50.1)	72 (44.7)	0.203
Age			
0–14 years	172 (18.8)	27 (16.8)	0.545
15–34 years	410 (44.8)	76 (47.2)	0.565
≥35 years	333 (36.4)	57 (35.4)	0.817
Unknown	1 (0.1)	1 (0.6)	
TB category			
I (any new case)	671 (73.3)	130 (80.7)	0.051
II (previously treated TB/TB meningitis)	135 (14.8)	14 (8.7)	0.039*
III (children under 8)	107 (11.7)	17 (10.6)	0.671
Unknown	3 (0.3)	0 (0)	
Pulmonary disease	650 (71.0)	120 (74.5)	0.601
HIV status			
Reactive	707 (77.4)	111 (68.9)	0.021*
Non-reactive	184 (20.2)	47 (29.2)	0.010*
Test not done	18 (2.0)	2 (1.2)	0.755
Unknown	4 (0.4)	1 (0.6)	
On HIV treatment	521 (73.7)	90 (81.1)	0.160

*p-value <0.05 i.e. statistically significant

Table 3 outlines treatment outcomes for the control and intervention groups. The intervention group had a higher chance of treatment success as compared with the control group: 72.7 % (117/161, 95 % CI 65.8–79.6 %) versus 60.4 % (553/916, 95 % CI 57.2–63.5 %), *p* = 0.003.

**Table 3:** Treatment outcomes for control and intervention groups

	Control *N*,% (95 % CI)	Intervention *N*,% (95 % CI)	*p*-value
Treatment success	553, 60.4 %	117, 72.7 %	0.003*
	(57.2–63.5 %)	(65.8–79.6 %)	
*Treatment completion*	423, 46.2 %	98, 60.9 %	0.001*
	(43.0–49.4 %)	(53.4–68.4 %)	
*Cure*	130, 14.2 %	19, 11.8 %	0.426
	(11.9–16.5 %)	(6.8–16.8 %)	
Incomplete treatment	339, 37.0 %	40, 24.8 %	0.003*
	(33.9–40.1 %)	(18.1–31.5 %)	
*Default*	98, 10.7 %	14, 8.7 %	0.443
	(8.7–12.7 %)	(4.3–13.1 %)	
*Death*	215, 23.5 %	17, 10.6 %	<0.001*
	(20.8–26.2 %)	(5.8–15.4 %)	
*Treatment failure*	26, 2.8 %	9, 5.6 %	0.069
	(1.7–3.9 %)	(2.0–9.2 %)	
Transferred out	24, 2.6 %	4, 2.5 %	1.000
	(1.6–3.6 %)	(0.1–4.9 %)	

* *p*-value <0.05 i.e. statistically significant

A summary of the logistic regression model, which the forward stepwise selection method generated, is provided in Table 4. The model produced a likelihood ratio test statistic of 1086.058 and a Nagelkerke’s R square value of 0.159. All selected covariates were significantly different from zero. The following covariates were selected for inclusion in the model: age, TB category, HIV positive status, whether the patient was on ART and whether the patient had a treatment supporter. All selected covariates were significantly different from zero (age, *p* = 0.000; TB category, *p* = 0.038; HIV positive status, *p* = 0.000; on ART, *p* = 0.000; treatment supporter, *p* = 0.08). Having an incentivised treatment supporter was shown to increase the chances of TB treatment success (OR = 1.826).

**Table 4:** Summary of the logistic regression model

Covariate	Coefficient	Standard error (coefficient)	Wald chi-square value	*p*-value	OR
Age (reference category 35+)			21.651	.000*	
0–14 years	2.081	.458	20.609	.000*	8.016
15–24 years	.273	.155	3.090	.079	1.314
TB (reference category 3)			6.546	.038*	
1	.843	.502	2.819	.093	2.324
2	.416	.527	.624	.430	1.516
HIV positive	−1.371	.241	32.249	.000*	.254
On ART	1.119	.181	38.177	.000*	3.061
Incentivised treatment supporter	.602	.227	7.018	.008*	1.826
Constant	-.011	.538	.000	.984	.990

* *p*-value <0.05 i.e. statistically significant

Incomplete treatment rates (including death, default and treatment failure) were significantly lower in the intervention group compared with the control (24.8 versus 37.0 %, *p* = 0.003). This was mainly due to the significant reduction in death in the intervention group compared with the control group: 10.6 % (17/161, 95 % CI 5.8–15.4 %) versus 23.5 % (215/916, 95 % CI 20.8–26.2 %), *p* = <0.001. There was no significant difference observed in patients defaulting (11 versus 9 %, *p* = 0.443).

## Discussion

This pragmatic controlled intervention study suggests that financial incentives for treatment supporters are beneficial in improving treatment outcomes. Patients who were supported by financially incentivised treatment supporters had higher rates of treatment success than those receiving standard care (72.7 % compared to 60.4 %). After adjusting for confounding factors (including age, sex and HIV status), patients who were supported by treatment supporters receiving financial incentives were 1.8 times more likely to complete treatment than those receiving standard care. In addition, the death rate was significantly reduced in the intervention group, compared with the control group, which may be clinically significant for a country with high mortality from TB. There is limited published evidence on incentives to improve TB treatment outcomes, with no decisive answer on whether they do in fact improve outcomes [4–8]. This paper adds to this literature from a variety of settings.

Improving TB treatment success is a priority in Swaziland, and in other countries with high burdens of TB and scarce resources. A number of interventions have been undertaken in Swaziland to reduce the burden of disease including household contact tracing [16], intensified case finding [17] and integrated HIV/TB community clinics [18]. Approaches to improving treatment outcomes may be of significant benefit to other high incident settings.

Incomplete TB treatment can lead to the development of drug resistance, which can lead to poorer health outcomes for the population. In addition, drug resistance is costly, as it often requires extended hospital admissions, more expensive drugs and long treatment periods [19]. Treatment of MDR TB in Southern Africa has cost over $15,000 per patient [20]. It is likely to be beneficial to invest in improving adherence and treatment success to prevent future costs and improve health.

There were a number of limitations to this study. The control group had significantly lower treatment success rates than the national figure. There is no clear explanation why this may be, but this does suggest underlying problems with local TB services and reflects regional differences in treatment outcomes. This difference in treatment success rates is not clearly explained in this study and will be further explored in the larger multi-centre study that is currently being undertaken. A 2004 study conducted in the area found a treatment success rate of 66 % in patients receiving family member treatment support [21]. As treatment success rates in this study were lower than expected, our previous sample size calculation was not valid. The revised power calculation found that the study was adequately powered at 80 % to detect a 12 % difference in treatment success from 60 to 72 %, with 900 controls and 130 in the intervention group.

This study only investigated incentives given directly to treatment supporters. The intervention was at the treatment supporter level, but only data on the treatment outcomes in the patients were collected, without an analysis of the impact of the characteristics on the treatment supporters themselves. This data were not routinely collected and therefore were not able to be presented. The study does not consider the impact of different methods of provision of financial incentives, for example comparing providing incentives to treatment supporters with providing financial support for the treatment itself, such as paying for transport or food costs. This would provide policy- and decision-makers with information on the different options for incentivising treatment support. The wider multi-centre study will investigate these differences.

A further limitation was that this was a pragmatic intervention study, and therefore randomisation of participants was not possible. Community support workers could only provide treatment support in the communities where they lived, which increased the risk of confounding factors influencing the study results. There was a significant difference in HIV status between the two groups, which suggests a sampling bias. The HIV population may have been provided with additional support through HIV treatment and care, which may also increase the compliance with TB treatment and treatment outcomes. This has been accounted for in the multiple logistic regression analysis. With this difference in HIV status adjusted for, patients supported by treatment supporters receiving financial incentives are 1.8 times more likely to complete treatment than those receiving standard care. The CSWs from the GSH live close to the hospital. This means that the intervention group included patients who lived in the area surrounding the hospital, while patients living outside the intervention area were included in the control group. As the study was not randomised, there may be an inherent difference between the intervention and control groups, as patients living further from the hospital may be poorer and more rural than those living nearer to the hospital. In particular, those living closer to the hospital might get better access to TB treatment, care and support. Lastly, the CSWs received some HIV and general training before the study began (not in TB), and therefore may have more skills in communication and health services than family members.

The control group received standard care, and support from an unpaid treatment supporter or family member. As this was a pragmatic study, it was not possible to investigate whether patients in the control group actually received support from their treatment supporter. This is one of the difficulties of relying on unpaid treatment supporters.

The study was conducted in a single treatment centre (GSH) in rural Swaziland and therefore these results may not be generalisable to the rest of Southern Africa. Further evaluation is required to assess the benefits in other centres, using a multi-centre trial, and will be included in the future study.

In this setting, incentives were provided through external funding from the Global Fund, through the National TB Programme. Sustaining funding for this project is important as financial incentives were found to be effective in improving treatment outcomes. This evidence will help to inform policymakers when developing TB support services in resource-limited settings.

## Conclusion

It appears that incentives provided to TB treatment supporters significantly improve TB treatment outcomes. There may be cost implications for TB programmes, however, incentivising treatment support may be appropriate as part of a comprehensive programme that moves towards achieving the international target of 85 % treatment success.

## Additional file

Additional file 1:
**Multilingual abstracts in the six official working languages of the United Nations.**

## Abbreviations

ART: Antiretroviral therapy
CBO: Community-based organisation
CSW: Community support worker
DOT: Directly observed therapy
Global Fund: The global fund to fight aids, tuberculosis and malaria
GSH: Good shepherd hospital
HIV: Human immunodeficiency virus
MDR: Multidrug resistance
TB: Tuberculosis
WHO: World health organisation
