# Supplementary material for: Effects of financial incentives for treatment supporters on tuberculosis treatment outcomes in Swaziland: a pragmatic interventional study
Source: Infect Dis Poverty. 2015 Jun 5;4:29. doi: 10.1186/s40249-015-0059-8 (PMC4456807; doi:10.1186/s40249-015-0059-8)
Supplement: Additional file 1: — Multilingual abstracts in the six official working languages of the United Nations. [file 40249_2015_59_MOESM1_ESM.pdf]

Translation of the abstract into the six official working languages of the United Nations

**أثر الحوافز المالية على مقدمي العلاج على نتائج علاج السل في سوازيلاند: دراسة تداخلية واقعية.**

كلينر م، مامفورا س، ندواندوي سز، بوسولوا ف، ويلفير و، ريتشاردسون م، والي ج، رايت ج.

**ملخص**

**خلفية:**

تعاني سوازيلاند من أعلى معدلات السل المستوطن في العالم، مع معدلات نجاح العلاج تصل لأقل بكثير من 85% من الهدف العالمي. العلاج المتاح جزء من خدمات السل الشاملة وهو مكون أساسي لاستراتيجية وقف السل. هذه الدراسة تبحث أثر الحوافز المالية على مقدمي العلاج على نتائج علاج السل في سوازيلاند.

**المنهج:** كانت الدراسة منضبطة تم فيها مقارنة نتائج العلاج للمرضى الذين تلقوا العلاج من معالجين حصلوا على حوافز مالية و آخرين لم يحصلوا على تلك الحوافز. **النتائج:** فرصة نجاح العلاج كانت أفضل في المجموعة الدخيلة بالمقارنة مع المجموعة المنضبطة: بواقع 73% (95% مجال الثقة 66% - 80%) للمجموعة الدخيلة، مقابل 60% (95% مجال الثقة 57% - 64%) للمجموعة المنضبطة، ب =0.003. وظل هذا التحسن كبيراً عند تغير معدلات نجاح العلاج لاختلفات أخرى في الصفات الأساسية، وكان تأثير الحوافز على مقدمي العلاج على العلاج نفسه بنسبة أرجحية تصل إلى 1.8. كان هناك أيضاً تحسن ملحوظ في معدل الوفيات في المجموعة الدخيلة بالمقارنة بالمجموعة المنضبطة (10.6% مقارنة ب23.5% ب=≤0.001)

**الاستنتاج:** يبدو أن الحوافز المعطاة لمقدمي علاج السل لها عامل كبير في التحسن الواضح لنتائج علاج السل. إعطاء الحوافز لمقدمي العلاج قد يكون ناجحاً كإضافة فعالة لتدابير الرقابة والدعم.

Translated from English version into Arabic by T. Catherine Hanna, through

TRANSLATORS
WITHOUT BORDERS

**干预性研究探索斯威士兰对结核病治疗效果支持者的财政激励效应**

Kliner M, Mamvura C, Ndwandwe SZ, Busulwa F, Welfare W, Richardson M, Walley J, Wright J

**摘要**

**引言：** 斯威士兰的结核病发病率全球最高，治疗成功率远低于 85%的国际目标。治疗支持作为结核病综合服务措施的一部分是遏制结核病策略的核心内容。本研究探索斯威士兰对结核病治疗效果支持者的财政激励效应。

**方法：** 通过对照试验，比较获得和未获得财政激励的治疗支持患者的治疗效果。

**结果：** 干预组治疗成功率 73%（95%置信区间：66%–80%）比对照组 60%（95%置信区间：57%–64%）的更高（*p*=0.003）。在矫正基线特征方面的差异后仍具有显著性差异，激励治疗支持者组的优势比为 1.8。此外，干预组在死亡率方面也有显著改善(10.6%比 23.5%，*p*=<0.001)。

**结论：** 研究表明对结核病治疗支持者的财政激励能显著改善治疗效果。这种激励措施可能是支持和监督措施的有效补充。

Translated from English version into Chinese by Yin Jian-hai, through

CHINESE CENTER FOR DISEASE CONTROL AND PREVENTION

NATIONAL INSTITUTE OF PARASITIC DISEASES

## Effets des incitations financières comme agents d'appui au traitement de la tuberculose au Swaziland: une étude interventionnelle internationale pragmatique

Kliner M, Mamvura C, Ndwandwe SZ, Busulwa F, Welfare W, Richardson M, Walley J, Wright J

**RESUME**

**Contexte:** Le Swaziland compte l'incidence nationale de tuberculose (TB) la plus élevée au monde, et les taux de succès du traitement sont bien en dessous de l'objectif international de 85 %. Un agent d'appui au traitement intégré à des services complets de lutte contre la tuberculose est un élément fondamental de la stratégie « Stop TB ». Cette étude analyse les effets des incitations financières pour les agents d'appui au traitement de la tuberculose au Swaziland.

**Méthodes:** Cette étude contrôlée a comparé les résultats des traitements pour des patients bénéficiant d'un agent d'appui ayant reçu ou non une incitation financière.

**Résultats:** Le groupe d'intervention a connu une plus grande chance de succès du traitement par rapport au groupe de contrôle: 73 % (95% des intervalles de confiance [CI] entre 66% et 80%) par opposition à 60% (95% des CI entre 57% et 64%), respectivement, *p*=0,003. Cette amélioration est restée substantielle lorsque les taux de succès du traitement ont été ajustés pour tenir compte des différences des caractéristiques de référence, ce qui a eu pour effet l'obtention d'un rapport de cote de 1,8 pour les résultats des traitements appuyés par des incitations. Le taux de mortalité a également connu une amélioration substantielle dans le groupe d'intervention par comparaison avec le groupe de contrôle (10,6% contre 23,5%, *p*=<0,001).

**Conclusion:** Les incitations fournies pour les agents d'appui au traitement de la tuberculose semblent améliorer les résultats du traitement de manière substantielle. Ajouter des incitations aux agents d'appui du traitement peut être un moyen efficace de prendre en charge et superviser les mesures.

Translated from English version into French by Michèle Landis, through

TRANSLATORS
WITHOUT BORDERS

## Как материальное поощрение помощников сказывается на результатах лечения туберкулеза в королевстве Свазиленд: исследование эффективности в условиях реальной клинической практики

Клинер М., Мамвура С., Ндвандве СЗ, Бусулва Ф., Велфэр В., Ричардсон М., Уолли Дж., Райт Дж.

**АННОТАЦИЯ**

**Предыстория:** В королевстве Свазиленд туберкулез (ТБ) получил более широкое распространение, чем в других странах мира, однако успешные результаты лечения составляют намного ниже планового международного показателя в 85%. Основной элемент стратегии борьбы с туберкулезом – это помощь в лечении в рамках комплексного обслуживания больных ТБ. Во время настоящего исследования изучено, как материальные поощрения помощников влияют на результаты лечения ТБ в королевстве Свазиленд.

**Методы:** Это было контролируемое исследование, в рамках которого сравнивались результаты лечения пациентов с помощниками, получившими и не получившими материальное поощрение.

**Результаты:** В экспериментальной группе вероятность успешного лечения оказалась выше, чем в контрольной группе: 73% (доверительный интервал при доверительной вероятности 0,95 [ДИ] 66%–80%) по сравнению с 60% (доверительный интервал при доверительной вероятности 0,95 [ДИ] 57%–64%), соответственно, $p$=0.003. Данное улучшение подтвердило свою значимость, когда показатели успешного лечения скорректировали на разницу в базовых характеристиках, при этом благоприятное влияние материально заинтересованных помощников на результаты лечения получило коэффициент 1,8. Кроме того, было замечено существенное уменьшение смертности в экспериментальной группе по сравнению с контрольной группой (10,6% против 23,5%, $p$=<0.001).

**Вывод:** Похоже, что поощрения помощников способствуют значительному улучшению результатов лечения ТБ, поэтому материальная заинтересованность может оказаться оптимальным дополнением к мерам поддержки и контроля над ходом лечения.

Translated from English version into Russian by Zoya Nayshtut, through

TRANSLATORS
WITHOUT BORDERS

**Efectos de los incentivos económicos a los protectores del tratamiento en los resultados del tratamiento contra la tuberculosis en Swazilandia: estudio pragmático de intervención**

Kliner M, Mamvura C, Ndwandwe SZ, Busulwa F, Welfare W, Richardson M, Walley J, Wright J

**RESUMEN**

**Antecedentes:** Swazilandia tiene la incidencia nacional de tuberculosis (TB) más elevada del mundo, con tasas de éxito del tratamiento bastante por debajo del 85% del objetivo internacional. El apoyo al tratamiento como parte de un servicio integral para la TB es un componente primordial de la estrategia Stop TB. El presente estudio ha investigado los efectos de los incentivos económicos a los protectores del tratamiento en los resultados del tratamiento contra la TB en Swazilandia.

**Métodos:** Estudio comparativo que ha comparado los resultados del tratamiento en pacientes con protectores del tratamiento que recibieron incentivos económicos y otros que no los recibieron.

**Resultados:** El grupo de intervención ha tenido más opciones de éxito en su tratamiento en comparación con el grupo de control. 73% (95% intervalo de confianza [CIs] 66%–80%) en contraposición a 60% (95% CIs

57%–64%), respectivamente, $p=0.003$. La mejora seguía siendo significativa al ajustarse las tasas de éxito por diferencias en características esenciales, lo que dio como resultado una razón de momios (RM) de 1,8 en los resultados de tratamientos con protectores de tratamiento incentivados. También hubo una mejora significativa en la mortalidad del grupo de intervención comparada con el grupo de control (10.6% en contraposición al 23.5%, $p=<0.001$).

**Conclusión:** Los incentivos ofrecidos a los protectores de los tratamientos contra la TB mejoran significativamente los resultados de los mismos. Incentivar el apoyo al tratamiento puede resultar conveniente como contribución eficaz a las medidas de apoyo y supervisión (199 palabras).

Translated from English version into Spanish by Raquel Bentué, through

TRANSLATORS
WITHOUT BORDERS
